# The type VII secretion system protects *Staphylococcus aureus* against antimicrobial host fatty acids

**DOI:** 10.1038/s41598-020-71653-z

**Published:** 2020-09-09

**Authors:** Arnaud Kengmo Tchoupa, Kate E. Watkins, Rebekah A. Jones, Agnès Kuroki, Mohammad Tauqeer Alam, Sebastien Perrier, Yin Chen, Meera Unnikrishnan

**Affiliations:** 1 Warwick Medical School, University of Warwick, Coventry, UK; 2 Department of Chemistry, University of Warwick, Coventry, UK; 3 Faculty of Pharmacy and Pharmaceutical Sciences, Monash University, Parkville, VIC 3052 Australia; 4 School of Life Sciences, University of Warwick, Coventry, UK

**Keywords:** Microbiology, Molecular biology

## Abstract

The *Staphylococcus aureus* type VII secretion system (T7SS) exports several proteins that are pivotal for bacterial virulence. The mechanisms underlying T7SS-mediated staphylococcal survival during infection nevertheless remain unclear. Here we report that *S. aureus* lacking T7SS components are more susceptible to host-derived antimicrobial fatty acids. Unsaturated fatty acids such as linoleic acid (LA) elicited an increased inhibition of *S. aureus* mutants lacking T7SS effectors EsxC, EsxA and EsxB, or the membrane-bound ATPase EssC, compared to the wild-type (WT). T7SS mutants generated in different *S. aureus s*train backgrounds also displayed an increased sensitivity to LA. Analysis of bacterial membrane lipid profiles revealed that the *esxC* mutant was less able to incorporate LA into its membrane phospholipids. Although the ability to bind labelled LA did not differ between the WT and mutant strains, LA induced more cell membrane damage in the T7SS mutants compared to the WT. Furthermore, proteomic analyses of WT and mutant cell fractions revealed that, in addition to compromising membranes, T7SS defects induce oxidative stress and hamper their response to LA challenge. Thus, our findings indicate that T7SS contribute to maintaining *S. aureus* membrane integrity and homeostasis when bacteria encounter antimicrobial fatty acids.

## Introduction

*Staphylococcus aureus* is a facultative pathogen that can colonize the skin and nares of healthy individuals. The asymptomatic carriage of *S. aureus* is a major risk for subsequent infections^1^. *S. aureus* infections, which can be healthcare or community-associated, range from benign impetigo to life-threatening bacteraemia^2^. Clinical management of staphylococcal infections is complicated by the increasing prevalence of multidrug resistant strains^3^.

The success of *S. aureus* as a deadly pathogen is attributed to an array of virulence factors that facilitate host tissue adhesion and immune response evasion^4^. One of these virulence factors is the type VII secretion system (T7SS), also known as the ESAT-6 secretion system (ESS). The orthologous ESX-1 system was initially discovered in *Mycobacterium tuberculosis*, where it is essential for bacterial virulence^5^. T7SSs (T7SSb) are found in both Gram-positive and Gram-negative bacteria, although these systems and their secretion machineries appear to be distinct to their mycobacterial counterparts^6^. In *S. aureus,* the T7SS displays modularity and heterogeneity in expression between different strains^7,8^. In extensively studied strains (COL, RN6390, USA300 and Newman), the T7SS consists of four integral membrane proteins (EsaA, EssA, EssB and EssC), two cytosolic proteins (EsaB and EsaG), five secreted substrates (EsxA, EsxB, EsxC, EsxD and EsaD), and EsaE, which interacts with the T7SS substrates to target them to the secretion apparatus^9^. A peptidoglycan hydrolase, EssH, was reported to mediate T7SS transport across the bacterial cell wall envelope^10^.

The molecular architecture of the staphylococcal T7SS has not yet been fully characterized. T7SS integral membrane proteins EsaA, EssA, EssB, and EssC are thought to be the core of the T7 secretion machinery, with EssC being the central membrane transporter^11–13^. Interactions between secreted substrates and co-dependent secretion of substrates have been demonstrated^7,9,14,15^. A recent study showed that the functional assembly of the T7SS machinery in *S. aureus* is supported by the flotillin homolog FloA, within functional membrane microdomains^16^.

The *S. aureus* T7SS is pivotal for bacterial virulence. Indeed, S. *aureus* mutants lacking the entire T7SS^7^ or specific T7SS components (EsxA, EssB, EssC, EsxC, EsxB, EsaB, EsaD or EsaE) were consistently shown to be less virulent and/or persistent in various mouse infection models^11,17–21^. EsxA is necessary to delay apoptosis of *S. aureus*-infected epithelial and dendritic cells, while other substrates modulate cytokine production^18,22,23^. Although the relevance of T7SS to *S. aureus* is less understood, a role for the toxin-antitoxin pair EsaD (or EssD) and EsaG (or EssI) was recently demonstrated in intraspecies competition^9,15^.

In the human host, *S. aureus* encounters fatty acids (FAs) in the blood, on the skin, the nasal mucosa and other lipid rich tissues^24–26^. Several unsaturated FAs, including palmitoleic acid (C16:1) and linoleic acid (C18:2) are known to inhibit *S. aureus* growth^27,28^. In mice, topical or intraperitoneal treatments with such antimicrobial FAs or diets rich in antimicrobial FAs decrease bacterial load and increase survival upon *S. aureus* infection^29,30^. Many saturated FAs like stearic acid, on the other hand, are non-toxic to this pathogen^31^. Irrespective of their saturation, host FAs can be incorporated into *S. aureus* phospholipids via fatty acid phosphorylation by a fatty acid kinase (Fak)^32^. It is thought that this incorporation may contribute to bacterial resistance against toxic host FAs^31^. Interestingly, *S. aureus* T7SS expression is induced in response to host-specific FAs^20,21,33^. The role of T7SS in bacterial resistance to antimicrobial FAs however remains unclear. In this study, we demonstrate that surprisingly, EsxC and other T7SS mutants were more sensitive to unsaturated FAs compared to the wild-type (WT). Although there were no differences in binding labelled linoleic acid (LA), LA induced a more leaky membrane in the T7SS mutants, and there was less incorporation of LA into EsxC mutant membrane phospholipids. Furthermore, cellular proteomics revealed that in addition to membrane discrepancies, T7SS mutants exhibited different redox and metabolic states, which likely resulted in a distinct response to LA.

## Results

### *S. aureus esxC* and *essC* mutants are more sensitive to antimicrobial fatty acids

**Figure 1:**
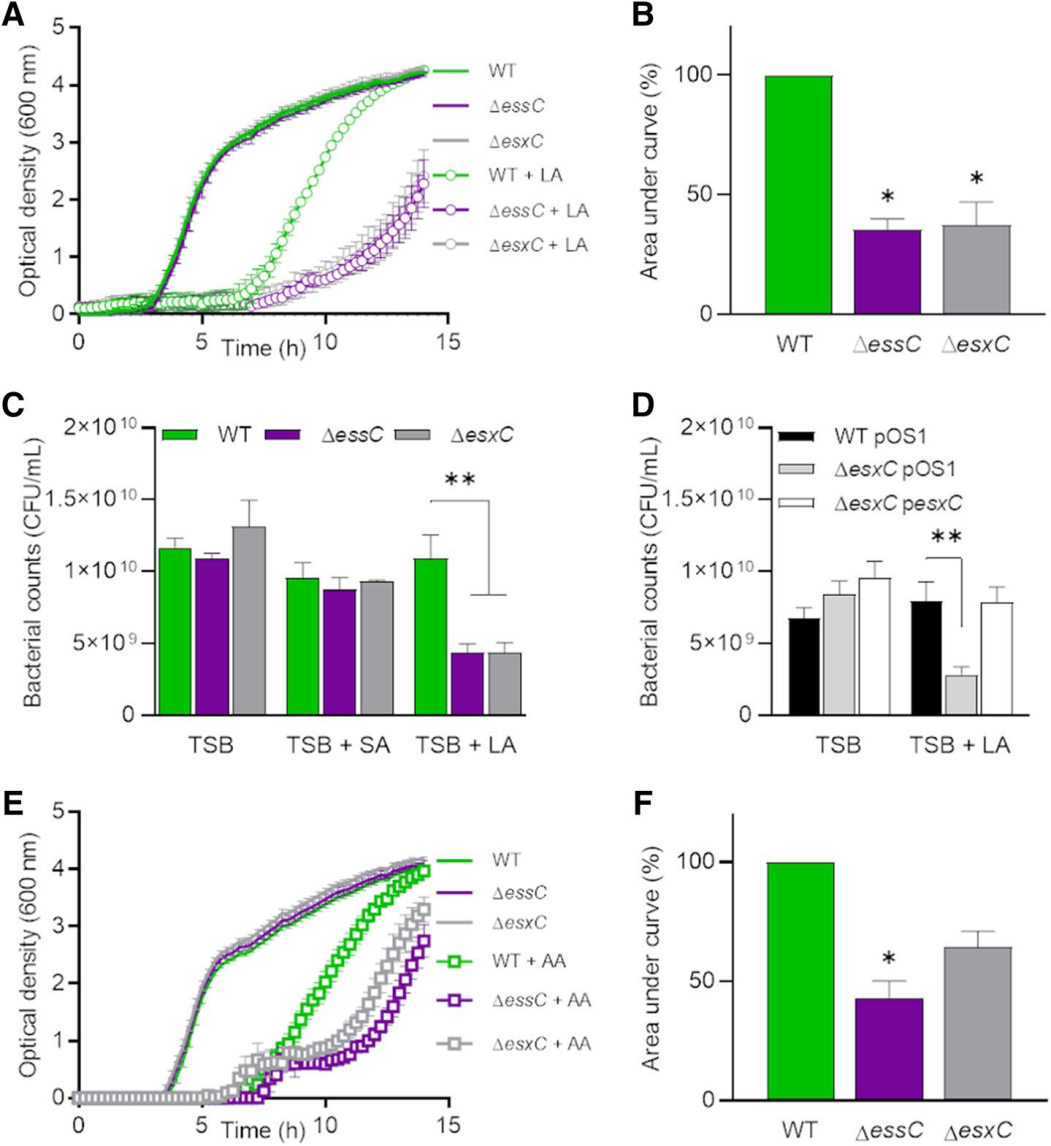
Enhanced *S. aureus* growth inhibition by antimicrobial fatty acids in *esxC* and *essC* mutants. (**A**) *S. aureus* WT USA300, Δ*essC*, and Δ*esxC* were grown in TSB or TSB supplemented with 80 µM linoleic (LA). Means ± standard error of the mean (SEM) are shown. *n* = 4. (**B**) The area under the curve (AUC) of biological replicates grown in TSB + LA in (A) were calculated and presented as % relative to the WT. Means ± SEM are shown. *Indicates *P* < 0.05 using a Kruskal–Wallis test with Dunn's multiple comparisons test. (**C**) After 14 h growth in TSB or TSB supplemented with 80 µM LA or stearic acid (SA), bacteria were serially diluted, and CFU were determined. Mean values are presented, and the error bars represent SEM. *n* = 3, **indicates *P* < 0.01 using one-way ANOVA with Dunnett’s test. (**D**) USA300 WT with the empty pOS1 plasmid (WT pOS1) and USA300 JE2 *esxC* mutant with either pOS1 (Δ*esxC* pOS1) or pOS1-*esxC* (Δ*esxC* pOS1-*esxC*) were grown in TSB or TSB + 80 µM LA as described in (A) followed by CFU estimation. Mean values are shown; error bars represent SEM. *n* = 5, **indicates *P* < 0.01 using one-way ANOVA with Dunnett’s test. (**E**) *S. aureus* WT USA300, Δ*essC*, and Δ*esxC* were grown in TSB or TSB supplemented with 80 µM arachidonic acid (AA). Means ± SEM are shown, *n* = 3. (**F**) AUCs of biological replicates grown in TSB + AA in (E) were calculated and presented as % relative to the WT. Means ± SEM are shown. *Indicates *P* < 0.05 using a Kruskal–Wallis test with Dunn's multiple comparisons test.

EsxC, a small 15-kDa protein secreted by the T7SS, is important for *S. aureus* persistence in mice^19^. However, mechanisms underlying EsxC- or T7SS-mediated bacterial survival are not known. In order to understand the role of EsxC, we generated an isogenic *esxC* mutant as described previously^34^, and the absence of any secondary site mutations was confirmed by whole genome sequencing. Δ*esxC* had a similar growth rate to the WT USA300 JE2 strain in standard rich medium (Supplementary Fig. S1). However, interestingly, when Δ*esxC* was cultured in the presence of an unsaturated FA (C18:2), linoleic acid (LA), at a concentration (80 µM) that still allows WT growth, it displayed significantly impaired growth, compared to the WT (Fig. 1A,B). Δ*esxC* did not show a growth defect when cultured in parallel in presence of stearic acid (SA), a saturated C18:0 FA (Supplementary Fig. S2A). A deletion mutant of the membrane-bound major ATPase EssC (a core T7SS component) also showed a similar growth defect in presence of LA but not SA. Both T7SS mutants displayed a decrease in optical density and colony forming units (CFU) in presence of LA but not SA (Fig. 1A,C, Supplementary Fig. S2A). Importantly, Δ*esxC* complemented with a plasmid containing the *esxC* gene reverted to the WT phenotype (Fig. 1D). The increased susceptibility of T7SS mutants to antimicrobial fatty acids was not restricted to linoleic acid as when cultured in the presence of arachidonic acid, another unsaturated FA (C20:4), growth of Δ*esxC* and Δ*essC* was inhibited more as compared to the WT (Fig. 1E, F).

### T7SS substrates contribute to *S. aureus* resistance to LA toxicity

**Figure 2:**
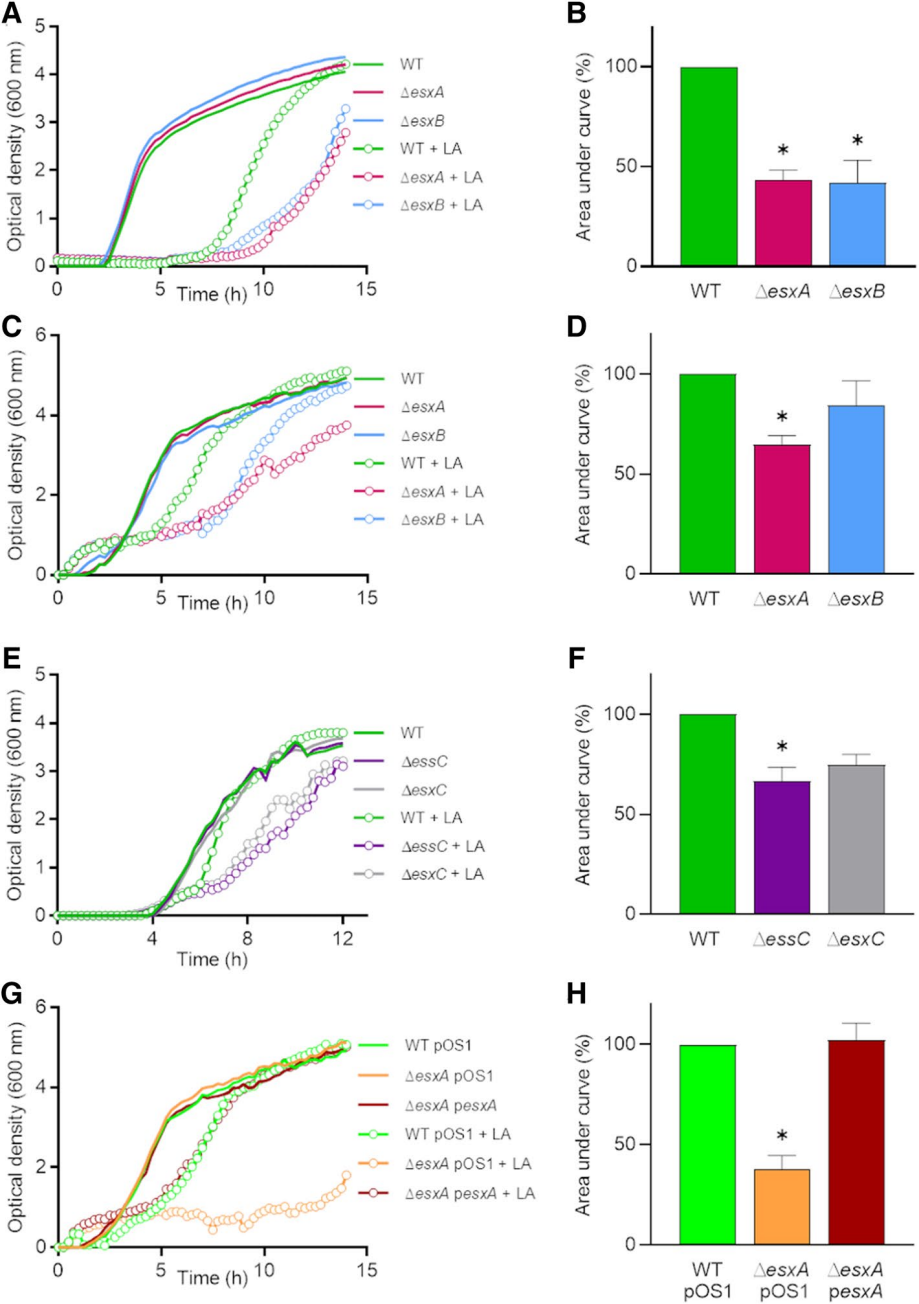
T7SS substrates contribute to resistance to linoleic acid toxicity. (**A**) *S. aureus* USA300 wild-type (WT) and USA300 *esxA* (Δ*esxA*) or *esxB* (Δ*esxB*) deletion mutants were grown in TSB or TSB supplemented with 80 µM linoleic acid (LA). (**B**) AUCs of biological replicates grown in TSB + LA as in (**A**) were calculated and presented as % relative to the WT. Means ± SEM are shown. *n* = 4. (**C**) *S. aureus* Newman WT and Newman *esxA* (Δ*esxA*) or *esxB* (Δ*esxB*) deletion mutants were grown in TSB or TSB + 40 µM LA. (**D**) AUCs of biological replicates grown in TSB + LA as in (**C**) were calculated and presented as % relative to the WT. Means ± SEM are shown. *n* = 4. (**E**) Growth curves as described in (**A**) were done with RN6390 wild-type (WT) and RN6390 *essC* (Δ*essC*) or *esxC* (Δ*esxC*) deletion mutants. (**F**) AUCs of biological replicates grown in TSB + LA as in (**E**) were calculated and presented as % relative to the WT. Means ± SEM are shown. *n* = 3. (**G**) Newman WT with the empty pOS1 plasmid (WT pOS1) and Newman *esxA* mutant with either pOS1 (Δ*esxA* pOS1) or pOS1-*esxA* (Δ*esxA* p*esxA*) were grown in TSB or TSB + 40 µM LA**.** Data shown in (**A**,**C**,**E**,**G**) are representative of at least three independent experiments. (**H**) AUCs of biological replicates grown in TSB + LA as in (**G**) were calculated and presented as % relative to the WT. Means ± SEM are shown. *n* = 4. In (**B**,**D**,**F**,**H**) *indicates *P* < 0.05 using a Kruskal–Wallis test with Dunn's multiple comparisons test.

Next, we investigated whether T7SS proteins other than *essC* and *esxC* contributed to *S. aureus* growth in presence of LA. Mutants lacking two other substrates, Δ*esxA* and Δ*esxB*, were grown in presence of FAs. Both mutants grew significantly slower than the WT USA300 LAC (Fig. 2A,B), in line with previous studies demonstrating the inter-dependency of the different T7SS substrates^7,14^. To ensure that the increased sensitivity observed for the T7SS mutants was not strain specific, RN6390 Δ*essC* or Δ*esxC* and Newman Δ*esxA* or Δ*esxB* mutants were tested. Similar to the USA300 mutants, the growth of all these T7SS mutants was also impacted in the presence of LA (Fig. 2C–F). Newman Δ*esxA*, and RN6390 Δ*essC* showed significantly decreased growth, while Newman Δ*esxB* and RN6390 Δ*esxC* had a slight growth defect. The growth defect in Newman Δ*esxA* was abrogated upon complementation (Fig. 2G,H). None of the T7SS mutants grew differently compared with WT in presence of stearic acid (Supplementary Fig.S2B–E). Of note, the Newman WT was readily inhibited by a lower concentration of LA (40 µM), which is in agreement with the lower T7SS expression levels in this strain compared to USA300^7,14^. We conclude that a functional T7SS plays a role in *S. aureus* resistance to LA toxicity.

### T7SS is required for maintaining membrane integrity in the presence of LA

**Figure 3:**
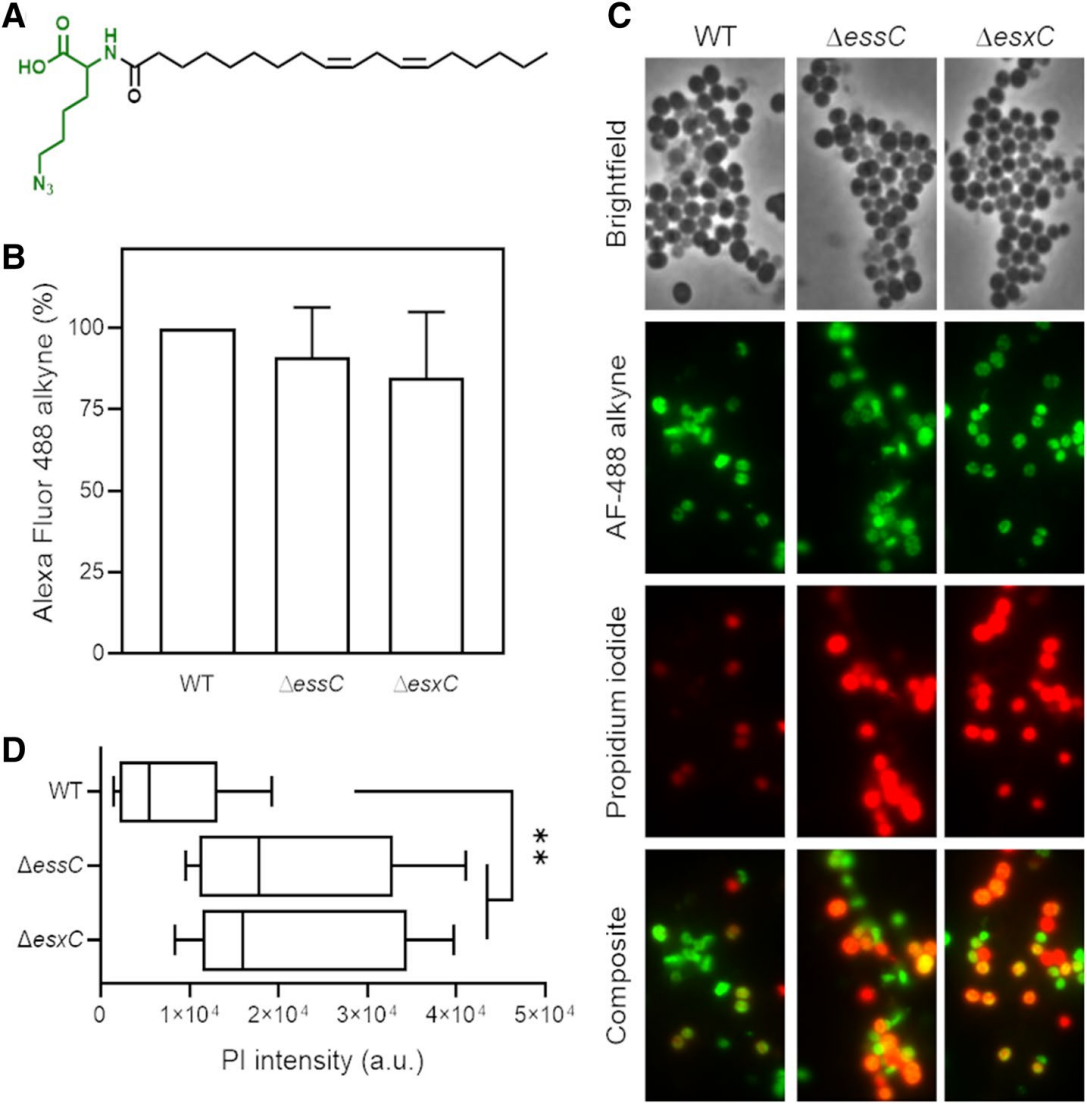
T7SS mutants display increased membrane permeability upon LA binding. (**A**) Chemical structure of azide functionalised linoleic acid (azide-LA; *N*^*6*^-diazo-*N*^*2*^-((9Z,12Z)-octadeca-9,12-dienoyl)lysine, N_3_-LA). Highlighted in green is the azido lysine. (**B**) *S. aureus* USA300 WT, Δ*essC*, and Δ*esxC* were grown with shaking in TSB to OD_600_ of 1.0. Bacteria were then stained for 15 min with 10 µM azide-LA prior to labelling for 1 h with alkyne Alexa Fluor 488. Mean percentage of fluorescence values relative to WT (100%) are presented; error bars represent SD, *n* = 5. (**C**) Micrographs of bacteria grown in TSB and treated as described in (**B**) and additionally stained with propidium iodide (PI). (**D**) ImageJ was used to quantitate PI fluorescence of bacterial clusters from 12 different fields per strain. Each box‐and‐whisker plot depicts the minimal and maximal PI intensities, the median is the vertical bar inside the box, which is delimited by the lower and upper quartiles. **Indicates *P* < 0.01 using one-way ANOVA with Dunnett’s test.

To study the mechanisms involved in T7SS mediated protection to LA toxicity, further studies were performed using mutants constructed in the USA300 JE2 strain lacking EsxC, a representative T7SS effector, or EssC the main T7SS transporter. To test if LA-mediated growth inhibition was due to an increased binding of LA to T7SS mutants, we chemically engineered LA to produce an azide functionalised LA (*N*^*6*^-diazo-*N*^*2*^-((9Z,12Z)-octadeca-9,12-dienoyl)lysine, N_3_-LA) or azide-LA (Fig. 3A). After incubating bacteria with azide-LA, click-chemistry with an alkyne dye (Click-iT Alexa Fluor 488 sDIBO alkyne) was used to stain azide-LA associated with bacteria. There were no obvious differences in the fluorescence from Δ*essC* and Δ*esxC* compared to the WT (Fig. 3B), suggesting that T7SS components are not involved in binding or sequestering LA.

**Figure 4:**
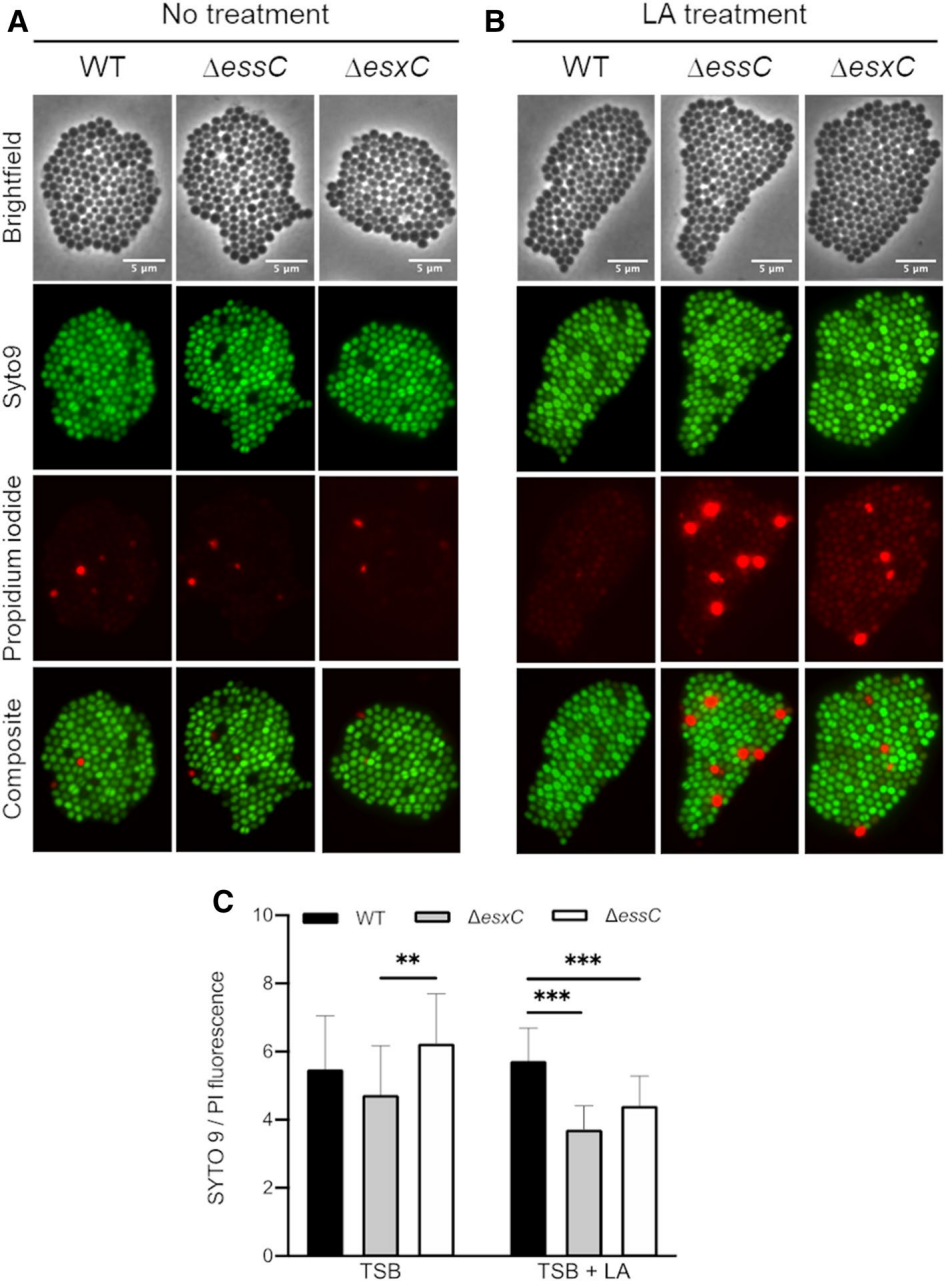
T7SS mutants display increased PI staining when treated with LA. Live/Dead staining of *S. aureus* USA300 WT, Δ*essC* or Δ*esxC* mutants after growth to OD_600_ of 1.0, without (**A**) or with treatment with 80 µM (**B**) linoleic acid. Images are representative of 3 independent experiments. (**C**) The ratio of SYTO 9: PI fluorescence (live:dead cells) of 25 different fields per strain was quantitated with ImageJ. Means ± SD are shown, *n* = 3; ***Indicates *P* < 0.001, **Indicates *P* < 0.01 using a one-way ANOVA with Tukey’s multiple-comparison test.

Unsaturated FAs have been well-documented to disrupt *S. aureus* membranes^31,35^. To study this, bacteria treated with azide-LA were also stained with propidium iodide (PI), a good indicator of membrane integrity. A more intense PI staining was observed for Δ*essC* and Δ*esxC* compared to the WT (Fig. 3C,D). Furthermore, to study the effects of unlabelled FA, WT and mutants were stained with PI and SYTO 9 after treatment with unlabelled LA. Again, an increased PI staining (Fig. 4A,B) and therefore lower SYTO 9/PI (Live/Dead) ratio was observed for both mutants (Fig. 4C). These data suggest that an intact T7SS helps *S. aureus* to maintain its membrane integrity when faced with the detergent-like effects of unsaturated FAs.

### LA-incorporation into membrane phospholipids is modulated by EsxC

**Figure 5:**
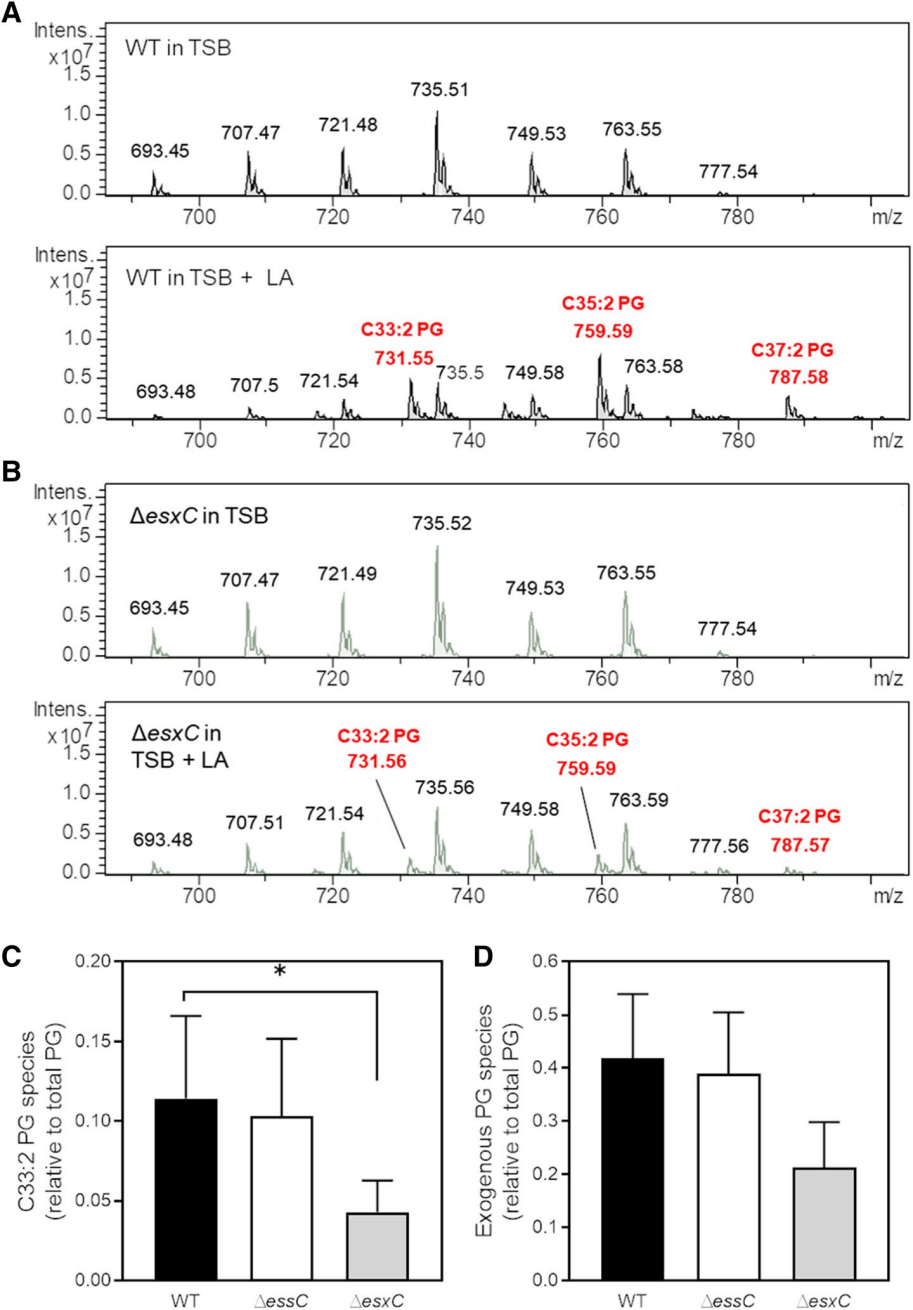
The *esxC* mutant is less able to incorporate LA into its phospholipids. Representative HPLC chromatograms of native phosphatidylglycerol (PG) species of *S. aureus* USA300 JE2 WT (**A**) or Δ*esxC* (**B**) grown in TSB (top panel) or in TSB supplemented with 10 µM LA (bottom panel), in negative ionisation mode. Relative quantification of the indicated PG species containing an unsaturated FA in LA-treated WT, Δ*essC* and Δ*esxC*. The C18:2-containing PG species, C33:2 (**C**) and total unsaturated fatty acid (C18:2, C20:2 and C22:2) containing exogenous PG species (C33:2, C35:2 and C37:2) (**D**) are presented as ratios of total PG species. Mean values are shown; error bars represent SD. *n* = 3, *indicates *P* < 0.05 using one-way ANOVA with Dunnett’s test.

When grown in presence of unsaturated fatty acids, *S. aureus* has been shown to incorporate unsaturated FAs into its membrane^31,36^. To investigate if membrane lipids were altered in the T7SS mutants, lipids from WT USA300 and T7SS mutants were analysed by high-performance liquid chromatography (HPLC)-mass spectrometry (MS) in negative ionisation mode. As reported previously^37,38^, phosphatidylglycerol (PG) was the major phospholipid present in the membrane of WT grown in TSB (Supplementary Fig. S3A). Δ*essC* and Δ*esxC* grown with or without 10 µM LA (a concentration that has been previously shown to be sub-inhibitory for USA300)^21^ displayed lipid profiles similar to that of WT (Supplementary Fig. S3A,B). Notably, PG molecular species were significantly altered upon growth in LA-supplemented TSB for WT (Fig. 5A), Δ*essC* (Supplementary Fig. S3C) and Δ*esxC* (Fig. 5B). Three new LA-specific PG species with mass to charge ratios (m/z) 731 (C33:2), 759 (C35:2), and 787 (C37:2) appeared to contain LA (C18:2) or its elongated C20:2 or C22:2 versions, as revealed by their fragmentations (Supplementary Fig. S4A–C). PG species containing exogenous, unsaturated FAs were also present in Δ*essC* and Δ*esxC*. However, LA (C18:2)-containing PG species (C33:2) were less abundant in the *esxC* mutant compared to WT (Fig. 5C). A similar trend, although statistically non-significant (*P* > 0.05), was observed for C20:2- and C22:2-containing PG species (Supplementary Fig. S4D,E), and when all the unsaturated exogenous PG species were combined (Fig. 5D). However, there were no significant differences in the incorporation of LA for Δ*essC* compared to WT. A possible explanation is that although Δ*essC* is defective in the secretion of EsxC, EsxC that accumulates in the cytosol^7,10^ and membranes (as indicated by our initial studies, Supplementary Fig. S5) of the Δ*essC* mutant, may mediate FA incorporation. Our data suggest that lack of the T7SS component EsxC may compromise the elongation and incorporation of LA into *S. aureus* phospholipids.

### T7SS mutations affect the total cellular content and *S. aureus* responses to LA

In order to gain further insight into T7SS-mediated modulation of proteins involved in FA incorporation and membrane homeostasis in presence of LA, we used an unbiased proteomic approach to study protein profiles of WT USA300, Δ*essC*, and Δ*esxC* grown exponentially with or without 10 µM LA. Of note, WT and both these T7SS mutants grew similarly in presence of up to 40 µM LA (Supplementary Fig. S6).

#### WT vs T7SS mutants in absence of LA treatment

**Figure 6:**
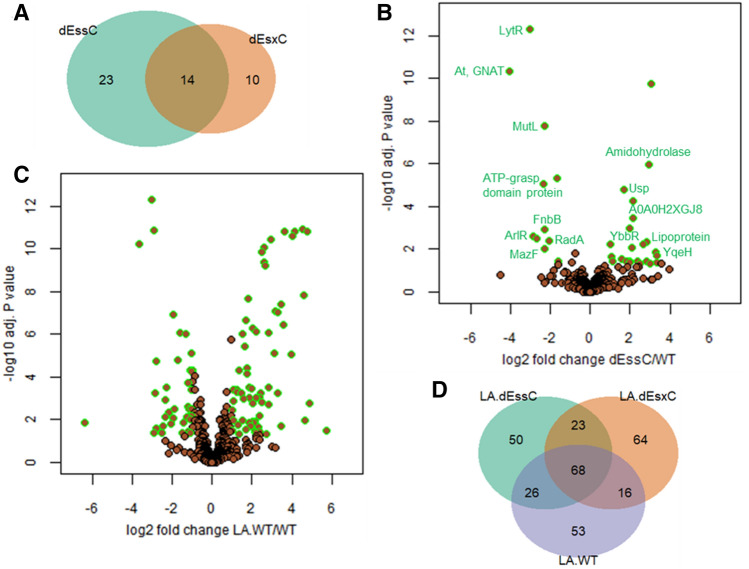
Quantitative proteomics shows altered cellular content and bacterial response to LA in T7SS mutants. *S. aureus* USA300 WT and mutants (Δ*essC* and Δ*esxC*) were grown in TSB or TSB supplemented with LA. (**A**) Venn diagram showing the number of proteins with altered abundance compared to WT specific to Δ*essC* (23) or Δ*esxC* (10), and common to Δ*essC* and Δ*esxC* (14). (**B**) The 14 proteins that are similarly changed in Δ*essC* and Δ*esxC* mutants are highlighted on a volcano plot. (**C**) Volcano plot showing the extensive change in the LA-treated WT compared to WT. (**D**) Venn diagram displaying the numbers of proteins with altered relative abundance upon LA challenge of WT (LA.WT), Δ*essC* (LA.dEssC) or Δ*esxC* (LA.dEsxC) compared to the respective untreated samples.

Interestingly, Δ*essC* or Δ*esxC* cultured in TSB readily displayed proteins with changed abundance when compared to the WT, with 37 and 24 proteins significantly (*P* < 0.05) altered in Δ*essC* and Δ*esxC*, respectively. Similarly, 14 proteins were differentially abundant in both Δ*essC* and Δ*esxC* (Fig. 6A,B). These included proteins associated with signal transduction (LytR and ArlR), the CW (acetyltransferase GNAT, FnbB and MazF), DNA repair (MutL and RadA), nucleotide binding (ATP-grasp domain protein and YqeH), hydrolysis (amidohydrolase), cell stress response [universal stress protein (Usp) family], or were uncharacterised (A0A0H2XGJ8, YbbR and lipoprotein) (Fig. 6B, Table 1). Of the 33 proteins changed only in Δ*essC* (23 proteins) or Δ*esxC* (10 proteins), nearly 40% (13 proteins) were associated with oxidation–reduction and other metabolic processes. Ten proteins which were annotated or reported to be membrane proteins, were more abundant in Δ*essC* (Table 1), which included SrrB, a membrane protein that is activated by impaired respiration^39^, and whose gene expression increased 6 times upon growth in presence of LA^21^. SrrB was also detected at higher levels in the *esxC* mutant although the increase was non-significant (*P* = 0.07).

**Table 1:** Proteins with changed abundance in Δ*essC* and Δ*esxC* mutants relative to the WT USA300 JE2.

Functions	Uniprot ID	Δ*essC*/WT		Δ*esxC*/WT		Description
		Log_2_ FC	Adjusted *P* value	Log_2_ FC	Adjusted *P* value	
Signal transduction systems	Q2FK09	− 3.0	4.90E−13	− 3.1	4.90E−13	Sensory transduction protein LytR
	Q2FH23	− 2.9	0.002527	− 2.1	0.026184	Response regulator ArlR
Membrane proteins	A0A0H2XF42	3.0	1.75E−10	0	1	Cytochrome D ubiquinol oxidase, subunit I
	A0A0H2XDZ5	1.7	1.68E−05	0	1	Uncharacterized membrane protein
	A0A0H2XFJ8	2.0	0.001077	0.5	0.883953	Uracil permease
	A0A0H2XGW7	2.7	0.005757	2.5	0.010784	Putative lipoprotein
	A0A0H2XIA9	1.0	0.006115	0	1	Protein translocase subunit SecY
	Q2FIN2	1.6	0.029451	0.5	0.970614	Prolipoprotein diacylglyceryl transferase LGT
	A0A0H2XKD9	2.0	0.038093	1.8	0.070347	Staphylococcal respiratory response protein SrrB
	A0A0H2XFE1	3.4	0.039814	1.1	0.970614	Peptidase
	A0A0H2XJV8	2.0	0.04444	1.5	0.231285	Cyclic-di-AMP phosphodiesterase
	A0A0H2XGF4	3.0	0.044681	0.9	0.986458	Sodium:dicarboxylate symporter family protein
	A0A0H2XHV2	2.5	0.049168	2.4	0.070182	Glycine betaine transporter OpuD
Stress response	A0A0H2XKH6	2.2	5.80E−05	2.5	1.07E−05	Universal stress protein family
	A0A0H2XIZ0	0.0	1	− 3.4	1. 06E−10	OsmC/Ohr family protein
DNA repair	Q2FHE2	− 2.3	1.79E−08	− 2.3	1.19E-08	DNA mismatch repair protein MutL
	A0A0H2XI63	− 2.0	0.004036	− 2.0	0.004036	DNA repair protein RadA
	A0A0H2XHT1	− 0.3	0.708343	− 1.9	0.008379	Formamidopyrimidine-DNA glycosylase MutM
Oxidation–reduction process	A0A0H2XJ90	1.1	0.038093	1.0	0.088334	D-isomer specific 2-hydroxyacid dehydrogenase family protein
	A0A0H2XHE0	2.4	0.039099	− 0.1	1	Thiol-disulphide oxidoreductase, DCC family protein
	A0A0H2XGR9	0.0	1	1.4	8.89E-08	Oxidoreductase, Gfo/Idh/MocA family
	A0A0H2XK08	1.0	0.406975	2.9	0.00791	Oxidoreductase, short chain dehydrogenase/reductase family
	A0A0H2XFZ3	− 0.8	0.404225	2.1	0.016405	Nitroreductase family protein
Hydrolases	A0A0H2XE49	2.9	1.07E−06	2.9	5.99E−07	Amidohydrolase
	Q2FES9	− 2.7	0.003385	− 0.3	1	Uncharacterized hydrolase
	A0A0H2XFF2	− 0.8	0.016697	− 0.5	0.157809	Peptidase, U32 family
	A0A0H2XJH8	0.0	1	2.8	1.06E−10	Peptidase M20 domain-containing protein 2
	A0A0H2XJ54	0.0	1	2.0	0.000949	Hydrolase (HAD superfamily)
	Q2FEG2	− 0.2	0.854748	− 2.9	0.004615	Formimidoylglutamase
Metabolism	A0A0H2XGU2	− 1.6	4.99E−06	− 0.1	1	Pseudouridine synthase
	A0A0H2XK15	2.8	0.004424	0.9	0.777138	1-phosphatidylinositol phosphodiesterase
	Q2FEK2	− 1.6	0.038093	− 0.3	1	Urease accessory protein UreE
	Q2FI05	1.1	0.038093	0.0	1	Bifunctional purine biosynthesis protein PurH
	Q2FIL2	2.9	0.038093	0.8	0.970614	SsrA-binding protein
	A0A0H2XII6	1.8	0.038093	1.5	0.088334	Orn/Lys/Arg decarboxylase
	A0A0H2XJR8	− 0.9	0.04444	− 0.5	0.61246	RNA methyltransferase, RsmD family
	A0A0H2XKG7	0.0	1	1.4	8.18E−08	Aspartokinase
Cell wall composition	A0A0H2XJQ4	− 4.0	4.92E−11	− 4.0	3.28E−11	Acetyltransferase, GNAT family
	A0A0H2XKG3	− 2.3	0.001238	− 1.7	0.016405	Fibronectin binding protein B
	A0A0H2XJC8	− 2.3	0.009779	− 3.0	0.000819	Phi77 ORF017-like protein (Toxin MazF)
	Q2FE03	0.0	1	2.6	1.18E−12	Fibronectin-binding protein A
Nucleotide binding	A0A0H2XHY5	− 2.3	8.58E−06	− 2.3	6.01E−06	ATP-grasp domain protein
	A0A0H2XFA5	3.3	0.014259	3.5	0.008379	Putative GTP-binding YqeH protein
Uncharacterised proteins	A0A0H2XGJ8	2.2	0.000372	2.4	9.41E−05	Uncharacterized protein
	A0A0H2XE09	2.1	0.008231	1.9	0.016405	Ybbr-like uncharacterized protein
	Q2FFI4	3.4	0.020136	0.9	0.970614	UPF0316 membrane protein
	A0A0H2XG24	1.1	0.022345	0.8	0.088334	Uncharacterized protein

#### WT vs T7SS mutants in presence of LA

We then compared the proteomic profiles of LA-treated strains (WT, Δ*essC* or Δ*esxC*) with their untreated counterparts. Clearly, the principal component analysis revealed that the differences due to the genetic makeup (WT or T7SS mutants) were less prominent than the dramatic changes induced by LA (Supplementary Fig. S7). These changes are exemplified for the WT; 163/1,132 proteins identified had an altered relative abundance upon growth with LA (Fig. 6C). 167 and 171 proteins were changed (*P* < 0.05) in Δ*essC* and Δ*esxC*, respectively, in response to LA, of which ~ 40% (68 proteins) were common to these mutants and their WT (Fig. 6D). At least 30% of proteins that were significantly different (*P* < 0.05) were unique to WT (53 proteins), Δ*essC* (50 proteins), or Δ*esxC* (64 proteins) (Fig. 6D), suggesting that each strain responds differently to LA. However, almost all proteins (13/14 proteins) that were similarly deregulated in Δ*essC* and Δ*esxC* grown without LA (Fig. 6B) were modulated in presence of LA (highlighted in bold in Dataset S1). Proteins that were less abundant in both mutants were, upon LA treatment, either increased to WT levels (MutL, acetyltransferase GNAT, Toxin MazF, and ATP-grasp domain protein), or were unchanged in the mutants and decreased in the LA-treated WT (LytR and FnbB) (Dataset S1). Likewise, proteins with increased abundance in Δ*essC* or Δ*esxC* were: (i) downregulated to WT levels (putative lipoprotein A0A0H2XGW7), (ii) unaltered in both mutants and upregulated in WT (Usp, amidohydrolase, and YbbR), (iii) or further increased in the *essC* mutant and strongly upregulated in WT (A0A0H2XGJ8) (Dataset S1). In sum, except for ArlR and RadA that were inversely regulated in all strains upon LA treatment, proteins similarly deregulated in Δ*esxC* and Δ*essC* were further modulated in response to LA, indicating that proteins altered by the lack of T7SS are important in the staphylococcal response to unsaturated fatty acids like LA.

#### Altered molecular functions in presence of LA

**Figure 7:**
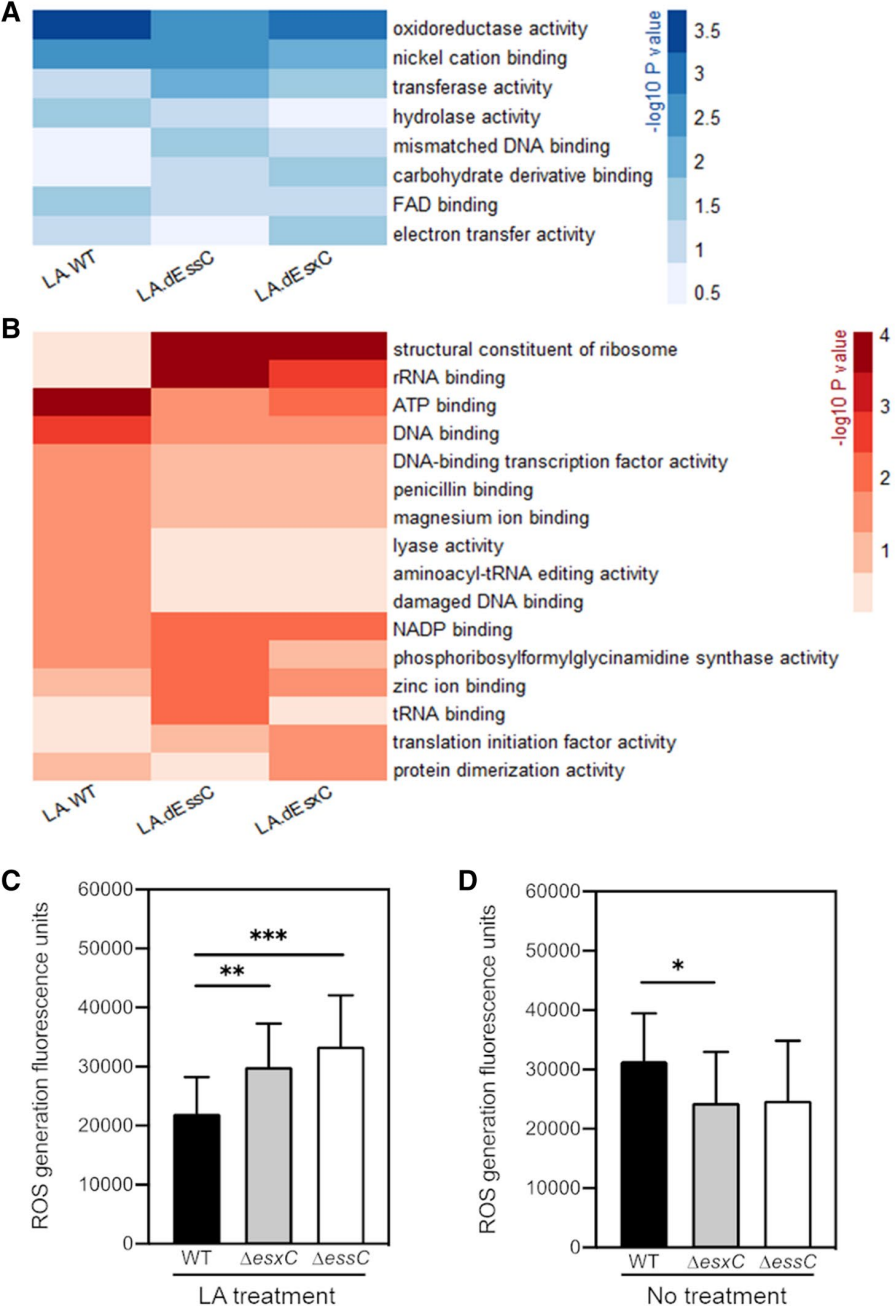
An altered oxidoreductive response in T7SS mutants in response to LA. Heatmaps depicting the *P* values of enriched (**A**) or diminished (**B**) molecular functions following a gene set analysis based on GO (gene ontology) annotations. Molecular functions that are changed in at least one strain (*P* < 0.05) following growth in presence of LA are shown. The shades of blue (**A**) or red (**B**) correspond to – log_10_ (*P* value). ROS levels were measured in cultures of *S. aureus* USA300 JE2 WT, Δ*essC* or Δ*esxC* grown to OD_600_ of 1.0 treated (**C**) with or without LA (**D**) using DCF reagent. Means ± SD are shown N = 5. *Indicates *P* < 0.05, **indicates *P* < 0.01, ***indicates *P* < 0.001 using the Kruskal–Wallis rank test.

We then used QuickGO (a web-based tool for Gene Ontology searching)^40^ to retrieve GO terms associated with the ten most significantly upregulated proteins in LA-treated WT (Dataset S1). Strikingly, 9/10 proteins had a hydrolase or an oxidoreductase activity. A comprehensive, statistical analysis showed a clear enrichment of 8 specific molecular functions (*P* < 0.05) in at least one strain (WT or T7SS mutants) (Fig. 7A). Oxidoreductase and hydrolase activities were enhanced in LA-treated WT, while Δ*essC* and Δ*esxC* were less able to upregulate proteins with these molecular functions. Flavin adenine dinucleotide (FAD)-binding, which plays a role in oxidation–reduction and FA metabolic processes, was similarly more enriched in the LA-treated WT. In contrast, transferase activity, which is linked to CW synthesis, was induced more in T7SS mutants compared to the WT. Molecular functions that are decreased upon LA challenge were also determined (Fig. 7B). In agreement with reduced intracellular ATP levels following membrane damage by antimicrobial FAs^35^, genes with the ATP-binding function (mainly ATP-binding ABC transporters) were negatively impacted in the WT. ATP-dependent lyases were also repressed in the WT. On the contrary, T7SS mutants were less able to modulate ATP-binding proteins. Instead, a strong inhibition of ribosomal constituents and other translation-related components was seen (Fig. 7B).

To test the oxidoreductive states of the WT and the mutants, we stained bacteria with dichlorofluorescin (DCF), which detects reactive oxygen species^41^. Reflecting the changes seen in the proteomics data, when treated with 10 µM LA there is an increase in the ROS generated in the T7SS mutants compared to the WT (Fig. 7C). However, in bacteria grown without LA, the mutants have slightly less or no change in the ROS generated compared to WT (Fig. 7D). Taken together, our proteomic analyses reveal that the lack of T7SS induces altered membrane and metabolic states indicative of oxidative stress responses. While multiple pathways are modulated in the WT to mitigate LA-induced damage on the bacterial membrane, such responses are clearly altered in the absence of the T7SS.

## Discussion

Host fatty acids (FAs) play a crucial role in the host defence to *S. aureus* infections. *S. aureus* is particularly sensitive to unsaturated FAs, which are abundant in the human skin^27,29,31,33,42^. We report here that the T7SS, an important component of *S. aureus* virulence arsenal, is critical in modulating the response to antimicrobial host FAs by maintaining the bacterial cell membrane integrity. A functional T7SS enables bacteria to mitigate LA-induced toxicity and grow better than mutants with a compromised T7SS. In the absence of T7SS components, LA is less incorporated into membrane phospholipids and enhances cell membrane damage. Furthermore, these bacteria are unable to activate adaptive mechanisms involved in LA resistance, as indicated by cellular proteomics.

T7SS loci in *S. aureus* vary in the organisation of four modules and their transcription across different strains. In this study, a protective role for the T7SS against FA toxicity was clear for *S. aureus* strains USA300, Newman, RN3690, which have a modular organisation representative of NCTC8325^8^. However, we see a variation in the degree of inhibition by LA with certain substrates and strains (Newman Δ*esxB* and RN6390 Δ*esxC*), which may be due to differences in T7SS expression and regulation between strains^7,14^. It is noteworthy that strains with other T7SS modular configurations have been reported to activate T7SS in response to LA (MRSA252)^33^ or require a functional T7SS for infection of FA-rich mouse skin (ST398)^43,44^. Hence, although T7SS substrates in many staphylococcal strains are yet to be characterised, it is plausible that the protective role of T7SS against toxic FA is conserved in strains with different T7SS. Additionally, several studies on the USA300 T7SS have shown multiple interactions between staphylococcal T7SS components, although the precise molecular architecture of this system remains unclear. EsxC (previously EsaC) was first described as a secreted protein^19^. However, it has been subsequently shown to localize within staphylococcal membranes^7,10^. Based on the available data, EsxC is likely to be associated to EsxA, EsaD, or EsaE on the membrane^9,14,18^.

The cellular proteomics data reveal that the abundance of more proteins is altered in Δ*essC* (37) than *esxC* (24) in comparison to *S. aureus* WT, which is in keeping with the greater importance of EssC as the conserved driving force of the T7SS^8^. Importantly, almost 60% of proteins deregulated in Δ*esxC* are similarly affected in Δ*essC*, strongly suggesting that any modification of the T7SS core leads to a similar staphylococcal response. Surprisingly, proteins with altered abundance in USA300 Δ*essC* were distinct to the ferric uptake regulator (Fur)-controlled genes differentially expressed in RN6390 Δ*essC*^45^. This discrepancy might be due to strain differences, including *rsbU* defect in RN6390 that impairs SigB activity^46,47^. Nevertheless, given the known role of Fur in oxidative stress resistance^48,49^, both mutants may display an altered oxidative status following *essC* deletion. *S aureus* RN6390 also differentially expresses redox-sensitive genes in absence of EsaB^50^. Also, since the T7SS substrate EsxA is upregulated in response to hydrogen pyroxide^45^, one could speculate that lack of T7SS stimulates an oxidative stress response. A further indication of altered physiological states of Δ*essC* and Δ*esxC* was the decreased abundance of the two-component regulatory system proteins, LytSR, ArlSR and SrrAB, which was consistent with down-regulation of *lytR* transcription observed previously in the absence of *arlR*^51^. Importantly, the *S. aureus* response to antimicrobial FAs includes downregulation of *lytRS*^33,52^, and upregulation of *srrB*^21^. Given that LytSR is involved in bacterial surface and membrane potential modulation^53,54^, T7SS defects are likely to result in an altered cell envelope.

It is striking that the staphylococcal T7SS is strongly upregulated in presence of sub-inhibitory concentrations of LA^21,33^. FAs with more cis double bonds, which are more toxic toward *S. aureus*^31^, are also more potent T7SS activators^21^. Our current study interestingly suggests a protective role of T7SS against LA toxicity. Previously described *S. aureus* antimicrobial FA resistance mechanisms, including IsdA or wall teichoic acid-mediated modulation of cellular hydrophobicity^29,31,55,56^, and FA detoxification with the efflux pumps Tet38 and FarE^57,58^, do not appear to explain the increased susceptibility of T7SS mutants to LA, as indicated by cellular proteomics. In line with a role for T7SS in the oxidative stress response, T7SS mutants were less able to prime their redox-active proteins in response to LA-induced oxidative stress. Instead, to cope with LA, they appear to rely on strong inhibition of the protein synthesis machinery, which is reminiscent of the stringent response^59^.

FA can inhibit *S. aureus* growth by destabilising the cell membrane through several mechanisms including membrane permeabilisation^31^. However, both toxic and non-toxic FA can be incorporated into bacterial phospholipids^31,60^, and reduced incorporation at inhibitory concentrations correlated with an accumulation of free FAs^31,36^. The incorporation of exogenous FA into membrane phospholipids occurs via a two-component fatty acid kinase (Fak)^31,32,60^, which was reported to be important for T7SS activation by unsaturated FA^21^. FakB1 and FakB2, bind to FAs, and FakB-bound FAs are phosphorylated by FakA prior to their incorporation^32^. Our lipidomic analyses revealed that in the absence of EsxC, bacteria were less able to incorporate LA into their phospholipids (Fig. 5), and displayed an increased membrane permeability in presence of LA. However, as all the T7SS mutants showed increased sensitivity to FA, it seems counterintuitive that LA incorporation was impacted more in Δ*esxC* than in Δ*essC* given the central role of EssC in T7 secretion^11–13^. In the incorporation experiments which were performed in presence of low non-inhibitory LA concentration (10 µM), it is possible that EsxC that accumulates in the membrane (Supplementary Fig. S5) in the absence of protein secretion by EssC, mediates LA incorporation in the *essC* mutant. In higher concentrations of LA, however, any T7SS defects may affect incorporation and hence sensitivity to unsaturated FA. It is also worth noting that transcript levels of *esxC*, and not *essC*, were strongly upregulated in a *S. aureus fakA* mutant^32^, Proteomic analyses however showed that protein levels of Fak proteins in the T7SS mutants remained unaltered in presence or absence of LA, suggesting no T7SS-mediated regulatory control of the Fak pathway. Although we currently do not understand the precise mechanisms involved in T7SS-mediated protection, we speculate that EsxC and other interdependent T7SS substrates may play a role in facilitating Fak function in *S. aureus* membranes, either by mediating recruitment or targeting of Fak proteins to the membrane. Further investigations are necessary to clarify the molecular mechanisms underlying T7SS-mediated FA incorporation within staphylococcal membranes.

The increased susceptibility of T7SS mutants to LA might explain why they are less virulent in environments rich in LA and other antimicrobial FAs, like the mouse lungs (Δ*essC*)^20^, abscesses (Δ*esxC* and Δ*esaB*), liver and skin (Δ*essB*)^21,44^. Previous research showing T7SS induction by host-derived FAs further supports the importance of T7SS in such environments^20,21^. Taken together, we conclude that T7SS plays a key role in modulating the *S. aureus* cell membrane in response to toxic host FAs. Although at present, it is unclear how T7SS contributes to staphylococcal membrane architecture, T7SS interaction with the flotillin homolog FloA within functional membrane microdomains^16^ corroborates the idea that T7SS proteins interact with many other proteins to modulate *S. aureus* membranes. Indeed, our data also suggest that blocking T7SS activity would make *S. aureus* more vulnerable to antimicrobial FAs, a key anti-staphylococcal host defence, thus making T7SS a very attractive drug target.

## Materials and methods

### Bacterial strains and growth conditions

The plasmid cured USA300 LAC JE2 strain and its mutants (Δ*essC* and Δ*esxC*) were used for most parts of the study. All *S. aureus* strains used are listed in Table S1, and were grown aerobically in tryptic soy broth (TSB) overnight (O/N) at 37ºC for each experiment unless stated otherwise. For complemented *S. aureus* strains, TSB was supplemented with 10 µg/mL chloramphenicol.

### Construction of bacterial mutants

The primers used are listed in Table S2. In-frame deletion of *essC* or *esxC* was performed as described previously^34^. Briefly, 1-kb DNA fragments up and downstream of the targeted gene sequence were PCR-amplified from USA300 LAC JE2 chromosomal DNA, and both PCR products fused via SOEing (splicing by overlap extension)-PCR. The 2-kb DNA fragment obtained was cloned into pKOR1, and used for in-frame deletion. Putative mutants were screened by PCR-amplification of a fragment including the gene of interest, whose deletion was confirmed by Sanger sequencing. Further, to confirm that successful mutants did not have any additional mutations, lllumina whole genome sequencing was performed on libraries prepared with the Nextera XT kit and an Illumina MiSeq instrument following manufacturers’ recommendations. For complementation, full-length *esxC* gene was cloned onto pOS1CK described previously^23^.

### Growth curves

O/N bacterial cultures were diluted to an OD_600_ of 0.05 in plain TSB or TSB supplemented with fatty acids. Bacteria were then grown in a 96-well plate with shaking, and the OD_600_ was measured every 15 min with a FLUOstar OMEGA plate reader (BMG Labtech, UK). Areas under the curves were computed with GraphPad Prism 8.0.

### Synthesis of azide functionalized linoleic acid

A 2-step synthesis was used to obtain *N*^*6*^-diazo-*N*^*2*^-((9Z,12Z)-octadeca-9,12-dienoyl)lysine, N_3_-LA (azide-LA). LA was first functionalized with *N*-hydroxysuccinimide (NHS) in anhydrous dimethyl formamide (DMF) in presence of *N*-(3-dimethylaminopropyl)-*N*’-ethylcarbodiimide hydrochloride. The solvent was then removed and replaced by dicholoromethane (DCM), following which the reaction mixture was washed with water and dried over magnesium sulphate. The product, 2,5-dioxopyrrolidin-1-yl (9Z,12Z)-octadeca-9,12-dienoate (NHS-LA), was analysed using ^1^H nuclear magnetic resonance (NMR) spectroscopy (Supplementary Fig. S8A) and mass spectrometry (MS). MS: [M + Na]^+^ 400.5 (calculated), 400.5 (found).
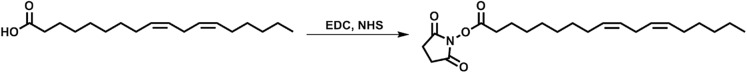


NHS-LA was left O/N at room temperature (RT) to react with l-azidolysine hydrochloride in anhydrous DMF, and produce azide-LA. 
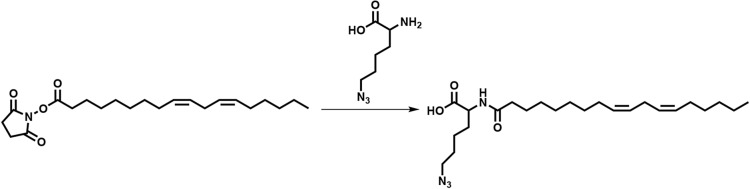


DMF was then removed, the reaction mixture precipitated in water, and dried under vacuum to obtain a clear oil. The composition of the oil was confirmed as being a mixture of azide-LA and unmodified LA (20% and 80%, respectively) based on ^1^H NMR (Supplementary Fig. S8B) and MS data. MS: [LA-H]^−^ 279.5 (calculated), 279.2 (found), [M-H]^−^ 433.3 (calculated), 433.6 (found).

### Binding assays with azide-LA and click chemistry

*S. aureus* USA300 JE2 WT, Δ*essC*, and Δ*esxC*, grown to OD_600_ of 1.0, were treated with 10 µM azide-LA for 15 min at 37 °C with shaking. The samples were then centrifuged, and the bacterial pellets resuspended in PBS supplemented with 4 µg/mL Click-iT Alexa Fluor 488 sDIBO alkyne (Life Technologies LTD, UK). After incubation at 25 °C for 1 h with shaking, bacteria were washed with PBS, and binding to azide-LA was quantified by measuring fluorescence using a FLUOstar OMEGA plate reader (BMG Labtech, UK). The samples imaged with a microscope were additionally stained with 3 µM propidium iodide, following click chemistry. Bacteria stained with Click-iT Alexa Fluor 488 sDIBO alkyne and 3 µM propidium iodide were immobilized on agarose-covered glass slides, and viewed with a Leica DMi8 widefield microscope (Leica Microsystems LTD, UK). Images were analysed with the ImageJ processing package Fiji^61^.

### Live/dead staining

Bacteria grown to OD_600_ of 1.0, were treated with 80 μM linoleic acid for 15 min at 37 °C with shaking. The samples were then centrifuged, and the bacterial pellets resuspended in PBS and supplemented with a 1:1 ratio of 2X LIVE/DEAD solution (6 μM SYTO-9 stain and 30 μM propidium iodide) from LIVE/DEAD BacLight kit (Invitrogen). After incubation in the dark for 15 min, bacteria were washed with PBS, spotted on to agarose pads and imaged using a Leica DMi8 widefield microscope (Leica Microsystems, UK). Acquired images were analysed with the ImageJ processing package, Fiji.

### Lipid extraction and analyses

Lipids were extracted from bacterial cultures as described elsewhere^62^. Briefly, bacteria were grown to OD_600_ of 1.0 in TSB or TSB supplemented with 10 µM LA, centrifuged in a 2 mL glass Chromacol vial (Thermo Scientific), and resuspended in 0.5 mL MS grade methanol (Sigma-Aldrich). MS grade chloroform was then used to extract lipids. The extracted lipids were dried under nitrogen gas with a Techne sample concentrator (Staffordshire, UK), and the lipid pellets resuspended in 1 mL acetonitrile. The samples were then analysed by LC–MS with a Dionex 3400RS HPLC coupled to an amaZon SL quadrupole ion trap mass spectrometer (Bruker Scientific) via an electrospray ionisation interface. Both positive and negative ionisation modes were used for sample analyses. The Bruker Compass software package was utilized for data analyses, using DataAnalysis for peak identification and characterization of lipid class, and QuantAnalysis for quantification of the relative abundance of distinct PG species to total PG species.

### Cellular proteomics

*S. aureus* strains were grown O/N at 37ºC on tryptic soy agar plates. The next day, single colonies were used to inoculate 10 mL plain TSB or TSB with 10 µM LA. Cultures were grown at 37ºC with 180-rpm shaking until an OD_600_ of 3.2 ± 0.2 was reached. The bacteria were then centrifuged, washed with PBS, and resuspended in lysis buffer (PBS, 250 mM sucrose, 1 mM EDTA, and 50 µg/mL lysostaphin) supplemented with cOmplete, mini EDTA-free protease inhibitor cocktail (Sigma-Aldrich, UK). After 15 min incubation at 37 °C, cells were lysed mechanically with silica spheres (Lysing Matrix B, Fischer Scientific, UK) in a fast-prep shaker as described previously^16^. Samples were then centrifuged, and the supernatants transferred to fresh tubes, where proteins were reduced and alkylated for 20 min at 70 °C with 10 mM TCEP (tris(2-carboxyethyl)phosphine) and 40 mM CAA (2-chloroacetamide), respectively. Next, the solvent was exchanged first to 8 M urea buffer then to 50 mM ammonium bicarbonate (ABC). Proteins were digested O/N at 37 °C with mass spectrometry grade lysyl endopeptidase LysC and sequencing grade modified trypsin (Promega LTD, UK).

### Label-free protein quantification

Peptides prepared for proteome analyses were desalted and concentrated with a C18 cartridge in 40 µL MS buffer (2% acetonitrile plus 0.1% trifluoroacetic acid). For each sample, 20 µL were analysed by nanoLC-ESI–MS/MS using the Ultimate 3000/Orbitrap Fusion instrumentation (Thermo Scientific), and a 90-min LC separation on a 50 cm column. The data were used to interrogate the Uniprot *Staphylococcus aureus* USA300 database UP000001939, and the common contaminant database from MaxQuant^63^. MaxQuant software was used for protein identification and quantification using default settings. Intensities were log_2_-tansformed with the Perseus software, and proteins with one or no valid value for every sample in triplicate were filtered. Missing values in cellular proteomics data were imputed on R. Specifically, for each sample, the imputed value was either the lowest intensity across all samples if at least two biological replicates had missing values or the average of two valid values if only one was missing.

### Immunoblotting

15 µg of proteins per sample (cell membrane and cell wall fractions) were loaded onto Mini-Protean TGX precast protein gels (Bio-Rad). After electrophoresis, proteins were transferred to PVDF membranes, which were cut and probed in parallel with anti-EsxC^7^ and anti-PBP2a^64^ antibodies.

### Statistical analyses

Except for the proteomics results, the statistical tests were performed with GraphPad Prism 8.0 as indicated in the Figure legends, with *P* values < 0.05 considered significant. A paired two-tailed Student’s t-test or a paired Mann–Whitney U test was used for pairwise comparisons. An ordinary one-way analysis of variance (ANOVA) with Dunnett's multiple comparisons test or a Kruskal–Wallis test with Dunn's multiple comparisons test was applied to data form three or more groups. The fold changes and *P* values of the proteomics data were calculated with the R package limma^66^, with USA300 JE2 WT or bacteria grown without LA as references.

## Supplementary information

Supplementary Information.

Supplementary Dataset S1.

## Data Availability

The mass spectrometry proteomics data have been deposited to the ProteomeXchange Consortium via the PRIDE^65^ partner repository with the dataset identifier PXD013081 and 10.6019/PXD013081. Cellular proteomic samples are labelled MS18-193.
