# Supplementary material for: The type VII secretion system protects Staphylococcus aureus against antimicrobial host fatty acids
Source: Sci Rep. 2020 Sep 9;10:14838. doi: 10.1038/s41598-020-71653-z (PMC7481793; doi:10.1038/s41598-020-71653-z)
Supplement: Supplementary file 1 — Supplementary Information. [file 41598_2020_71653_MOESM1_ESM.pdf]

The type VII secretion system protects *Staphylococcus aureus* against antimicrobial host fatty acids

Arnaud Kengmo Tchoupa[1], Kate E. Watkins[1], Rebekah A. Jones[1], Agnès Kuroki[2], Mohammad Tauqeer Alam[1], Sebastien Perrier[1,2,3], Yin Chen[4], Meera Unnikrishnan[1*]

[1]Warwick Medical School, University of Warwick, Coventry, United Kingdom

[2]Department of Chemistry, University of Warwick, Coventry, United Kingdom

[3]Faculty of Pharmacy and Pharmaceutical Sciences, Monash University, Parkville, Victoria 3052, Australia

[4]School of Life Sciences, University of Warwick, Coventry, United Kingdom

Running title: *S. aureus* T7SS and fatty acid toxicity

[*]Corresponding author

Email: M.Unnikrishnan@warwick.ac.uk.

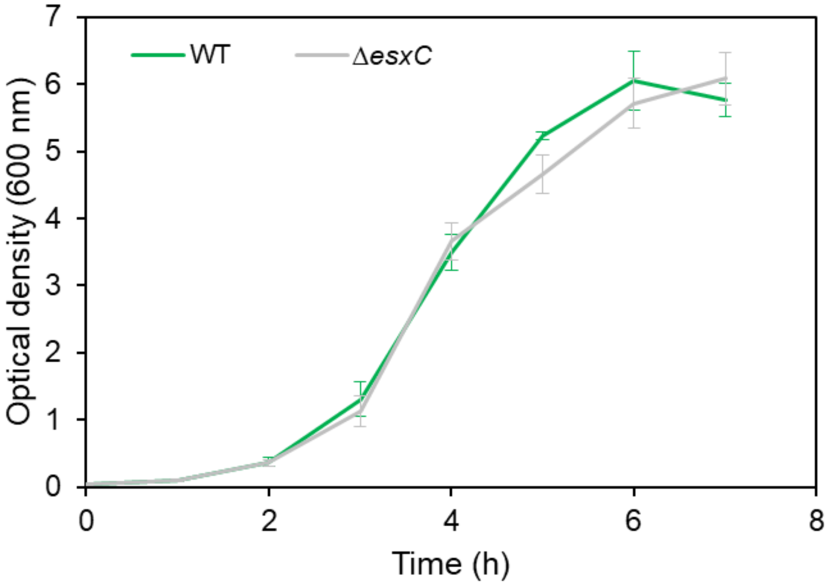

Fig. S1. USA300 JE2 WT and Δ*esxC* strains display similar growth rates. WT and Δ*esxC* were grown in TSB, and OD_{600} monitored with a Novaspec Pro spectrophotometer. Data shown are means of three independent experiments, and the error bars indicate the standard errors of the mean.

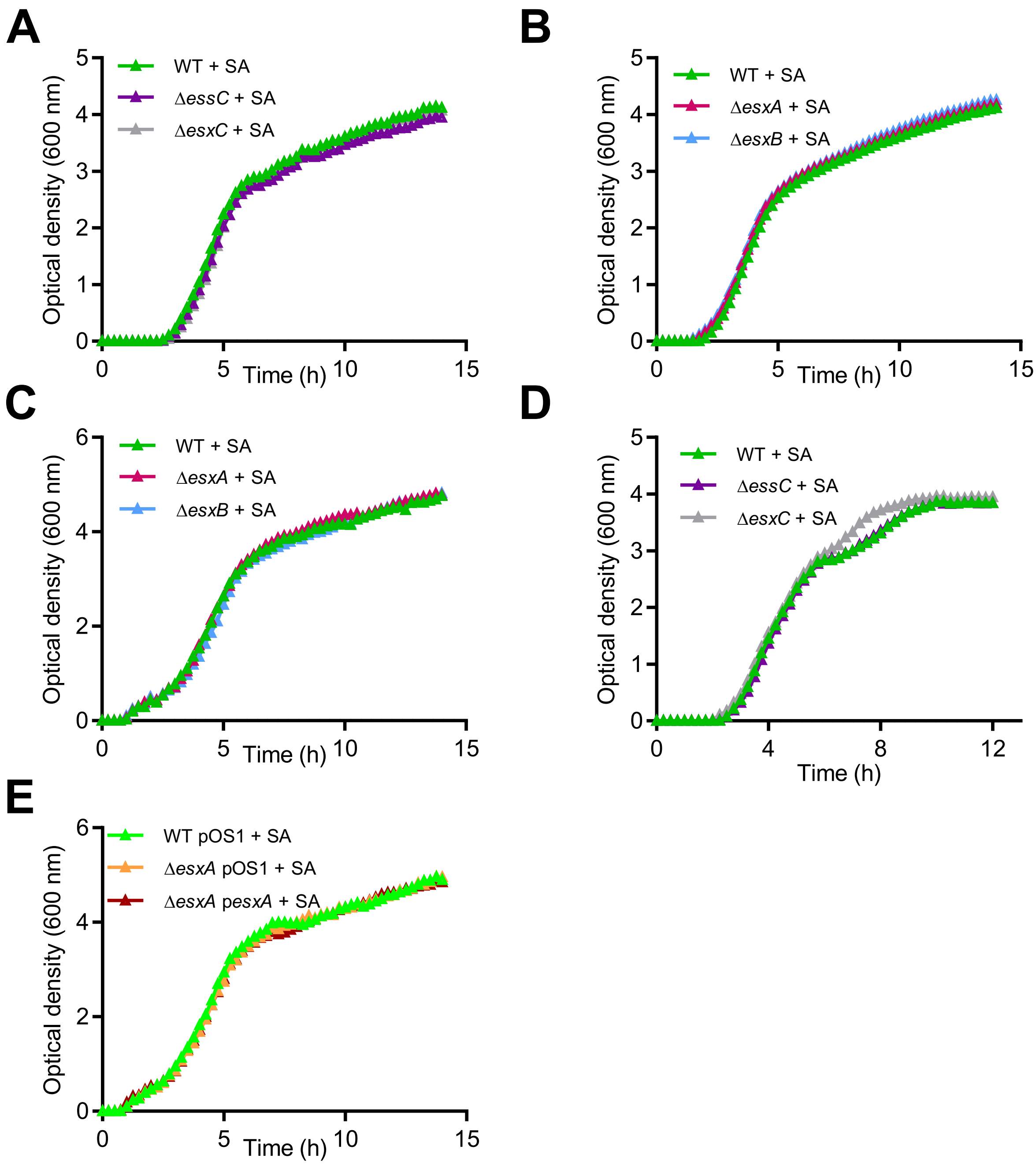

Fig. S2. T7SS mutants are not inhibited by stearic acid. The following *S. aureus* T7SS mutants and their respective WT strains were grown in TSB or TSB supplemented with stearic acid (SA): (A) USA300 JE2 WT, Δ*essC*, and Δ*esxC*; (B) USA300 LAC WT, Δ*esxA*, and Δ*esxB;* (C) Newman WT, Δ*esxA*, and Δ*esxB;* (D) RN6390 WT, Δ*essC*, and Δ*esxC*; (E) Newman WT with the empty pOS1 plasmid (WT pOS1) and Newman *esxA* mutant with either pOS1 (Δ*esxA* pOS1) or pOS1-*esxA* (Δ*esxA* p*esxA*). Data shown are representative of at least three independent experiments.

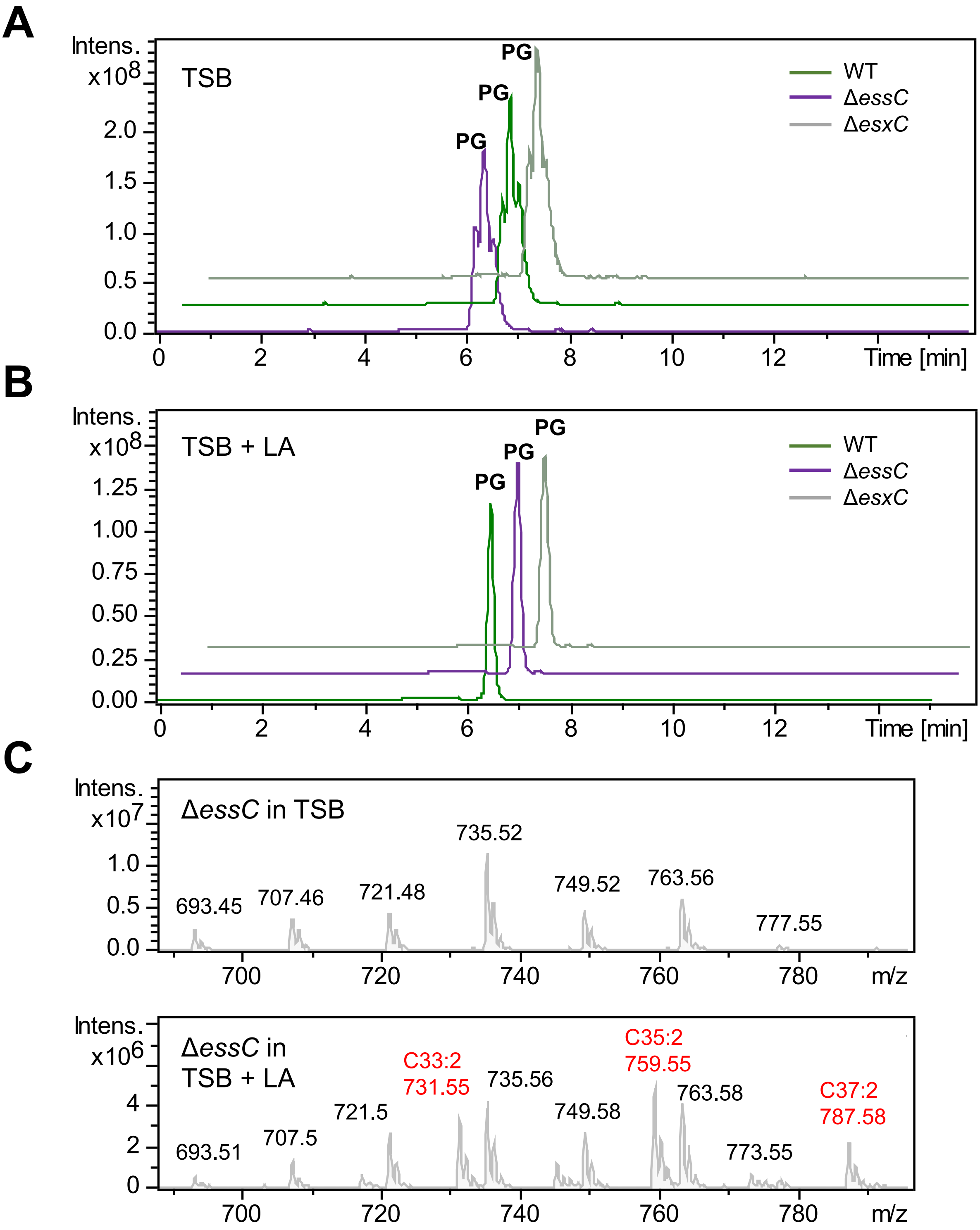

Fig. S3. USA300 JE2 WT and T7SS mutants display similar lipids. Representative HPLC chromatograms of the indicated bacteria grown in TSB (A) or in TSB supplemented with LA (B), in negative ionisation mode. Phosphatidylglycerol (PG) is highlighted.C. Representative HPLC chromatograms of native PG species of Δ*essC* grown in TSB (top panel) or in TSB supplemented with LA (bottom panel), in negative ionisation mode.

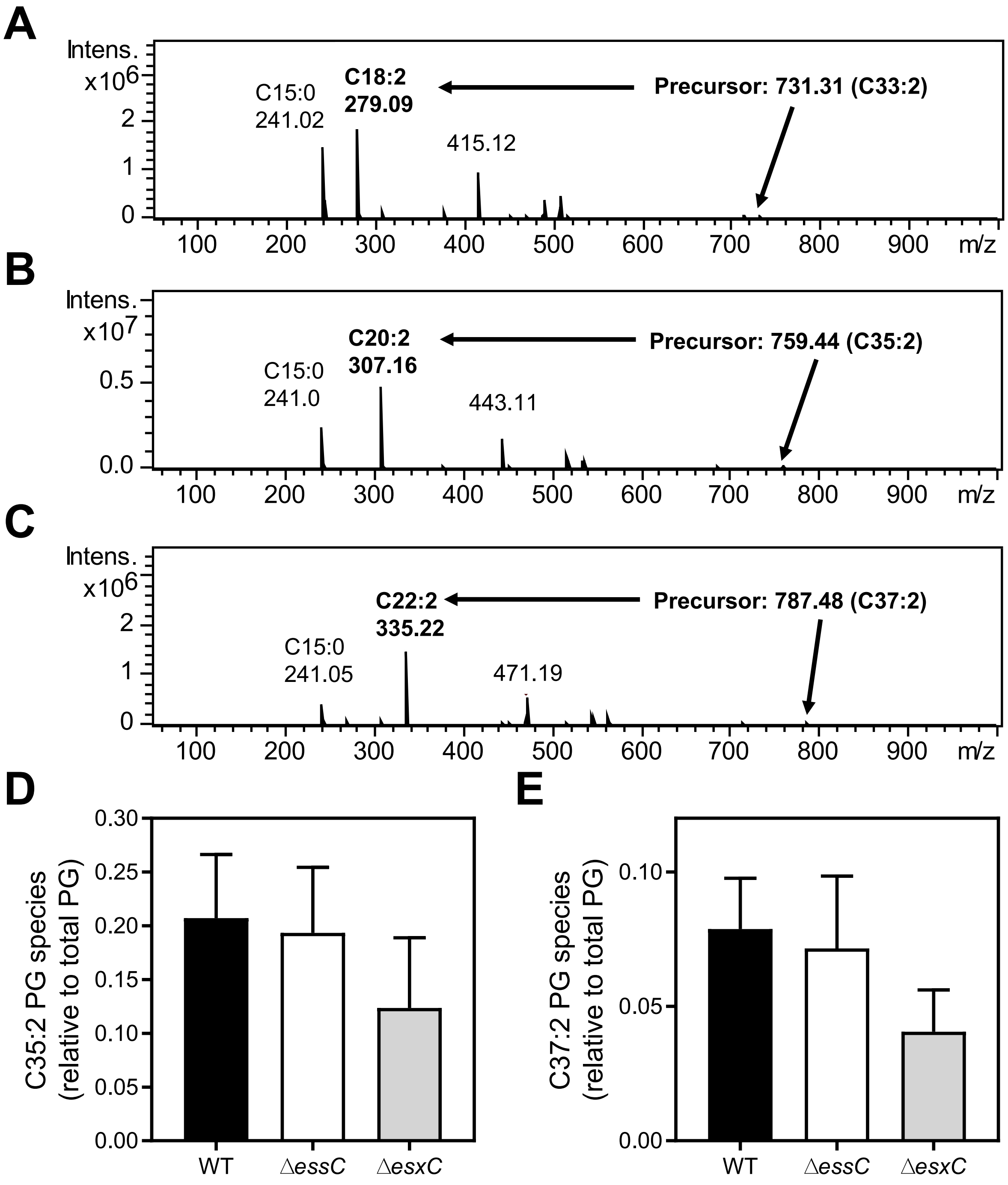

Fig. S4. LA (C18:2) is elongated and incorporated into *S. aureus* phosphatidylglycerol (PG) species. A-C. Representative mass spectrometry fragmentation spectra for PG species containing unsaturated fatty acids, in negative ionisation mode.

A) PG species with mass 731 m/z, containing C18:2 fatty acid (279 m/z).

B) PG species 759 m/z, containing C20:2 fatty acid (307 m/z)

C) PG species 787 m/z, containing C22:2 fatty acid (335 m/z).

D) and E. Relative quantification of the indicated PG species containing an unsaturated fatty acid in WT, Δ*essC* and Δ*esxC*. C20:2- (D) and C22:2-containing PG species (E) are presented as ratios of total PG species. Data shown are the means and error bars represent SD of three independent experiments.

A

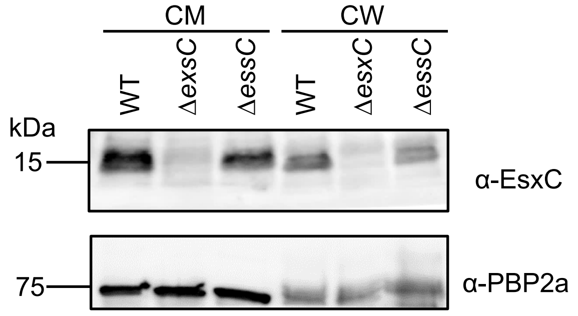

B

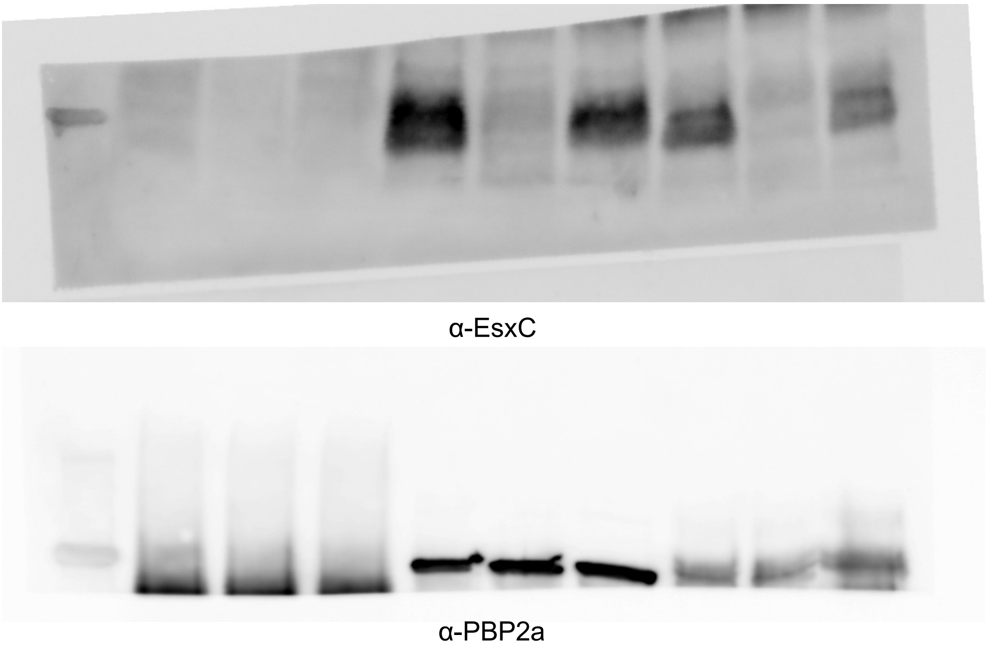

Fig. S5. Localisation of EsxC in *S. aureus* WT and Δ*essC* mutants. A) Immunoblot analysis of cell membrane (CM) or cell wall (CW) fractions of WT (USA300), Δ*essC*, and Δ*esxC* with anti-EsxC sera or anti-PBP2a antibodies (loading control). The images have been cropped for clarity. After sample transfer, the blots were cut and probed in parallel with anti-EsxC and anti-PBP2a antibodies (loading control). B) Images of unprocessed blots shown in A.

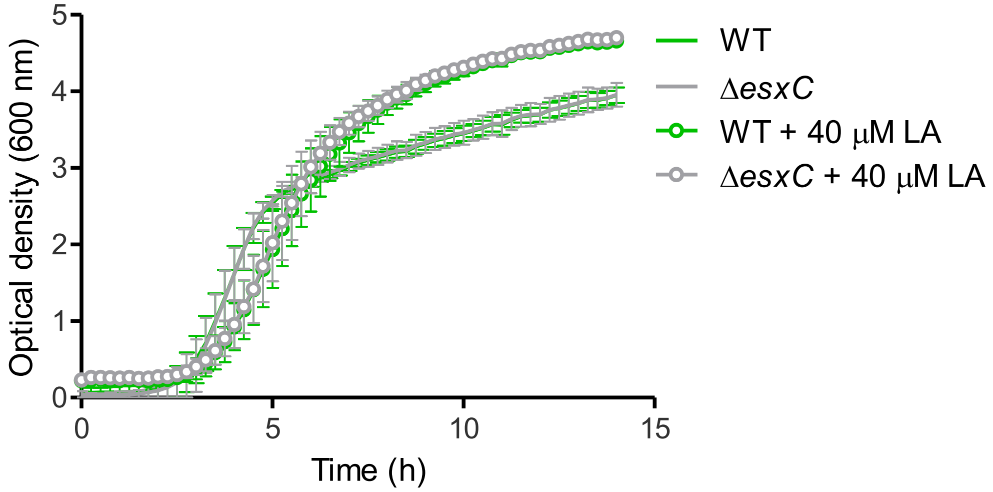

Fig. S6. USA300 JE2 WT and Δ*esxC* growth similarly in presence of sub-inhibitory levels of linoleic acid. *S. aureus* WT USA300 and Δ*esxC* were grown in TSB or TSB supplemented with 40 μM linoleic acid (LA). Means ± standard error of the mean (SEM) are shown, *n* = 3

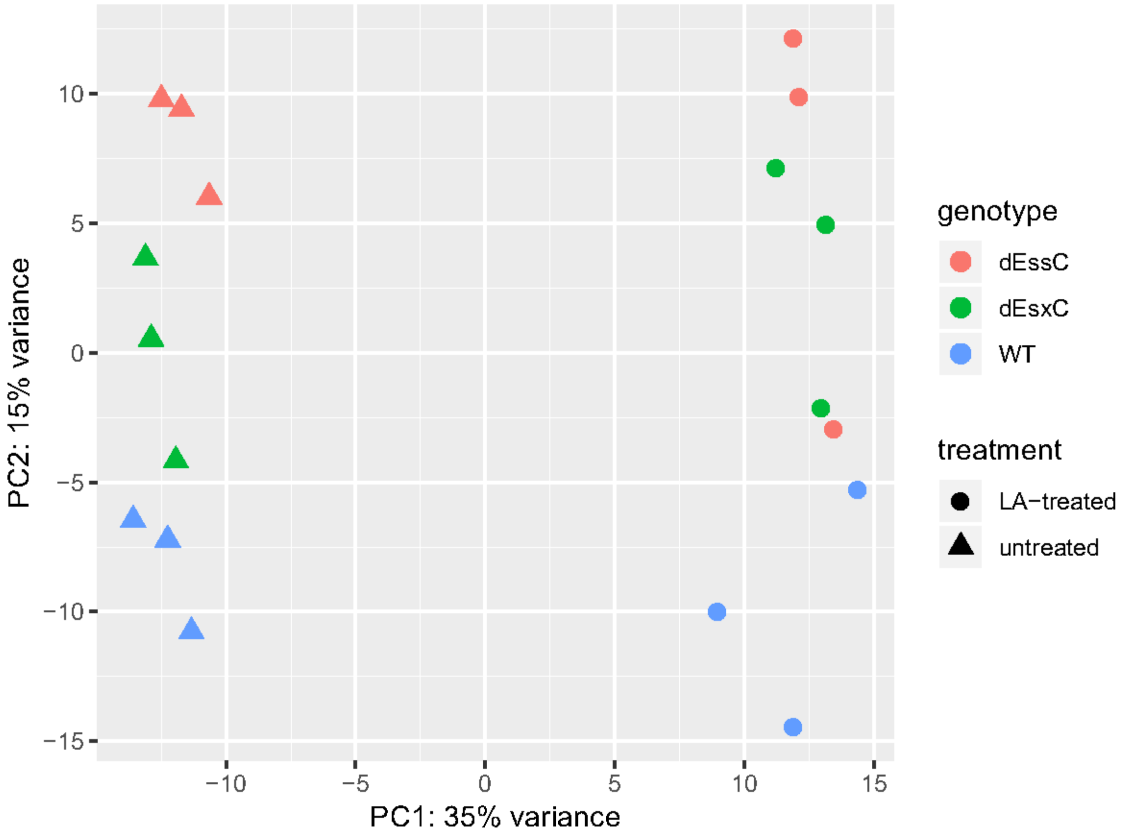

Fig. S7. **Principal component analysis (PCA) of the *S. aureus* cellular proteomic profiles.** PCA was performed on all the identified proteins of USA300 JE2 WT and T7SS mutants grown in TSB (untreated) or TSB + LA (LA-treated). Each dot represents a biological replicate.

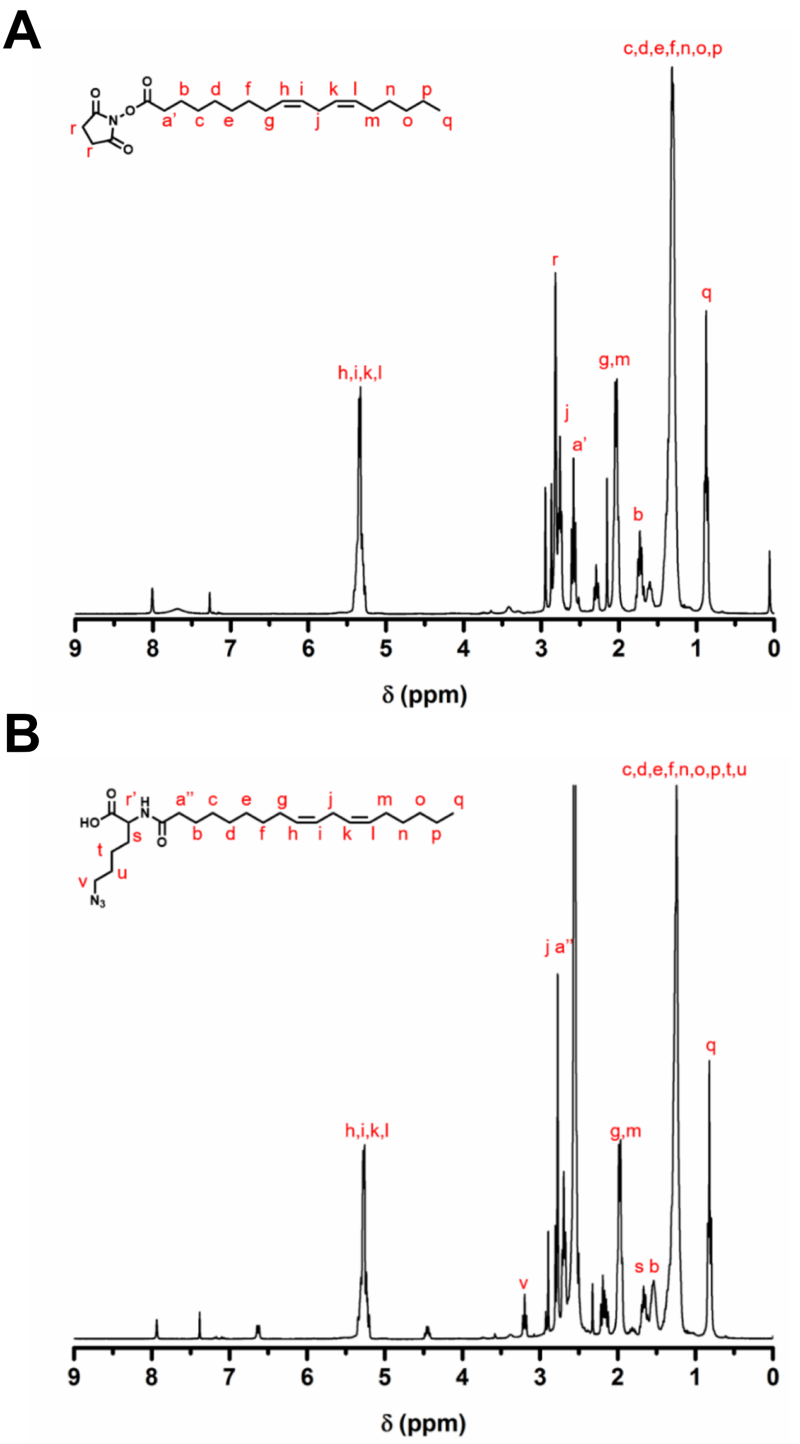

Fig. S8. ^{1}H NMR spectra of NHS-LA (A) and azide-LA (B) in $CDCl_3$. Both spectra were recorded on a Bruker Advance 300 spectrometer (300 MHz) at 27 °C. The letters indicate the chemical shift δ (in parts per million, ppm) of the protons in each molecule.

Table S1. Strains and plasmids used in this study.

Strain or plasmid	Description	Source or reference
Staphylococcus aureus		
USA300 LAC	Community-acquired MRSA (CA-MRSA)	Olaf Schneewind
LAC Δ*esxA*	*S. aureus* USA300 LAC defective for EsxA	(1)
LAC Δ*esxB*	*S. aureus* USA300 LAC defective for EsxB	(1)
USA300 LAC JE2	Plasmid-cured USA300 LAC	BEI Resources (NARSA) (2)
JE2 Δ*esxC*	*S. aureus* USA300 LAC JE2 defective for EsxC	This study
JE2 Δ*essC*	*S. aureus* USA300 LAC JE2 defective for EssC	This study
Newman	Methicillin-sensitive *Staphylococcus aureus*	Olaf Schneewind
Newman Δ*esxA*	*S. aureus* Newman defective for EsxA	(1)
Newman Δ*esxB*	*S. aureus* Newman defective for EsxB	(1)
RN6390	NCTC8325 derivative, Δ*rbsU*, Δ*tcaR*, cured of φ11, φ12, and φ13	Tracy Palmer (3)
RN6390 Δ*esxC*	*S. aureus* RN6390 defective for EsxC	Tracy Palmer (3)
RN6390 Δ*essC*	*S. aureus* RN6390 defective for EssC	Tracy Palmer (3)
RN4220	*S. aureus* restriction negative, cloning tool	BEI Resources (NARSA)
**Plasmids**		
pKORI	Temperature-sensitive allelic exchange vector	Olaf Schneewind (4)
pKORIΔ*essC*	pKORI used to generate *essC* mutant	This study
pKORIΔ*esxC*	pKORI used to generate *esxC* mutant	This study
pOS1	Insertless vector for genetic complementation	Olaf Schneewind
pOS1CK	pOS1 with P1 constitutive promoter of *sarA*	(1)
pOS1-*esxA*	*esxA* complementation vector	(1)
pOS1-*esxC*	*esxC* complementation vector	This study

Table S2. Primers used in this study.

Primer	Sequence (5' – 3')	Product
AttB1-*esxC* Up-Fwrd	GGGGACAAGTTTGTACAAAAAAGCAGGCTGAGCTAACGCTATGAAAACACC	AttB1-Δ*esxC*-AttB2
esxC-up-Rev-soeing	ACCCATATCTTCACCTCAATAAACATACCTCCCTCCTATTT	
esxC-down-Fwd	TATTGAGGTGAAGATATGGGTGG	
esxC-down-Rev-attB2	GGGGACCACTTTGTACAAGAAAGCTGGGTCGTCATTACTCCTCTGCTTTA	
AttB1-*essC*-up-fwd	GGGGACAAGTTTGTACAAAAAAGCAGGCTGCTACACATTTGTGTTGGCACC	AttB1-Δ*essC*-AttB2
*ess*C-upstream-rev	TGTCTTTGCCTCAGTCCTATAC	
essC-dpwn-soeing-fwd	GTATAGGACTGAGGCAAAGACACAATGAATTAAATAGGAGGGAGG	
esxC-down-Rev-attB2	GGGGACCACTTTGTACAAGAAAGCTGGGTCGTCATTACTCCTCTGCTTTA	
esxC-RBS-PstI-fwd	GCGCTGCAGTTGAGAGGAGAGAAAATGAATTTTAATGATATTGAAAC	RBS-*esxC*
esxC-SmaI-rev	GCGGCGCCCGGGTTAATTCATTGCTTTATTAAA	

Supplemental references

1. Korea CG, Balsamo G, Pezzicoli A, Merakou C, Tavarini S, Bagnoli F, Serruto D, Unnikrishnan M. 2014. Staphylococcal Esx proteins modulate apoptosis and release of intracellular Staphylococcus aureus during infection in epithelial cells. Infect Immun 82:4144-53.
2. Fey PD, Endres JL, Yajjala VK, Widhelm TJ, Boissy RJ, Bose JL, Bayles KW. 2013. A genetic resource for rapid and comprehensive phenotype screening of nonessential Staphylococcus aureus genes. MBio 4:e00537-12.
3. Kneuper H, Cao ZP, Twomey KB, Zoltner M, Jager F, Cargill JS, Chalmers J, van der Kooi-Pol MM, van Dijl JM, Ryan RP, Hunter WN, Palmer T. 2014. Heterogeneity in ess transcriptional organization and variable contribution of the Ess/Type VII protein secretion system to virulence across closely related Staphylocccus aureus strains. Mol Microbiol 93:928-43.
4. Bae T, Schneewind O. 2006. Allelic replacement in Staphylococcus aureus with inducible counter-selection. Plasmid 55:58-63.
